# Efficacy and tolerability of telaprevir (TVR)-based triple therapy in HIV/HCV-coinfected patients

**DOI:** 10.7448/IAS.17.4.19633

**Published:** 2014-11-02

**Authors:** Ignacio De Los Santos, Marisa Montes, Jose Sanz-Moreno, Julio De Miguel, Jesus Sanz-Sanz, Gabriel Gaspar, Laura Perez-Caballero

**Affiliations:** 1 Internal Medicine, Hospital Universitario De La Princesa, Madrid, Spain; 2 Internal Medicine, Hospital Universitario de la Paz, Madrid, Spain; 3 Internal Medicine, Hospital Universitario Principe de Asturias, Alcala de Henares, Spain; 4 Internal Medicine, Hospital Universitario de Getafe, Getafe, Spain

## Abstract

**Introduction:**

Clinical trials (CT) on triple therapy against HCV infection in HIV-infected patients including TVR plus pegylated interferon and ribavirin (PR) have reported considerably higher response rates than with PR alone. This study was aimed to evaluate the efficacy and safety of triple therapy including TVR in HIV/HCV-coinfected patients in real-life conditions.

**Materials and Methods:**

HIV/HCV genotype 1 patients seen at four Hospitals in Madrid who received therapy including TVR plus PR for at least two weeks were included. The response was evaluated during treatment, and sustained viral response (SVR) was evaluated 12 and 24 weeks after the end of the treatment.

**Results:**

Fifty-eight patients have been included; 79% male, median age 48 y.o.; 38% were IL28B rs12979860 genotype CT or TT, 58.6% of patients presented cirrhosis and 24.1% presented fibrosis F3. Infection with genotype 1a was observed in 53.4% of patients. Median baseline HCV-RNA was 3,282,263 IU/mL (77.5% had >800,000 IU/mL). The most commonly used antiretroviral (ARV) drugs were tenofovir/emtricitabine [36 (62%) patients], etravirine [21 (36%) patients], abacavir/lamivudine [18 (31%) patients], boosted protease inhibitors [16 (27.5%) patients] and raltegravir [12 (20.6%) patients]. Of the 42 (72.4%) patients who had received previous HCV treatment, 13.7% were null responders, 25.8% were partial responders and 31% had relapsed. In an ITT approach, proportions of patients with undetectable HCV RNA were 67.8% (38/56) at TW4, 83.3% (40/48) at TW12, 80% (36/45) at TW24, 79.4% at TW36 (31/39) and 72% (26/36) at TW48. Fifteen (25.8%) patients discontinued HCV therapy [8 (13.8%) because they fulfilled stopping rules, 5 (8.6%) individuals due to adverse events and 2 (3.4%) were lost to follow-up]. Rash associated with TVR (grade 1) was observed in two cases (3.4%) and all the patients showed anaemia at some point of treatment. In an analysis by ITT in the 31 patients who had a 60 week follow-up after starting therapy, SVR-12 was observed in 21 (67.7%) patients. And in the analysis by ITT in 28 patients who had a 72 week follow-up after starting therapy, SVR-24 was observed in 17 (60.7%) patients.

**Conclusions:**

Response to triple therapy with TVR plus PR in HIV/HCV-patients under real-life conditions, and therefore, including a high proportion of difficult to treat patients, is similar to that found in CT. The safety profile of TVR-based therapy is also comparable to that shown in CT, with only a rate of discontinuation of 8.6% of individuals related to toxicity.

